# Prenatal diagnosis of a maternal 7.22-Mb deletion at chromosome 4q32.2q32.3 by SNP array

**DOI:** 10.1186/s13039-020-00480-8

**Published:** 2020-04-10

**Authors:** Pingping Zhang, Yanmei Sun, Ping Huo, Haishen Tian, Jian Gao, Yali Li

**Affiliations:** Department of Reproductive Genetic Family, Hebei General Hospital, No.348 West Heping Road, Xinhua District, Shijiazhuang, Hebei Province People’s Republic of China 050051

**Keywords:** Variant of unknown significance, Deletion of 4q32.2q32.3, Chromosomal microarray analysis, Genetic counselling

## Abstract

**Background:**

Although Chromosomal microarray analysis (CMA) is a powerful diagnostic technology for detecting chromosomal copy number variants (CNVs), it detects numerous variants of unknown significance (VUSs), which poses a great challenge for genetic counselling. Terminal deletion of the long arm of chromosome 4 is a rare genetic aberration. Few cases of interstitial deletion sharing the common deleted segment have been reported.

**Case presentation:**

A male foetus with a 7.22-Mb deletion at chromosome 4q32.2q32.3 was found in the proband. The paternal genotype was normal. His asymptomatic mother with a normal phenotype and intelligence was found to carry the same deletion at the long arm of chromosome 4. The clinical significance of arr[GRCh37] 4q32.2q32.3(162858958_170081268)×1 remains uncertain. To the best of our knowledge, this is the first case report on a VUS of 4q32 deletion and the second report of a heterochromatic CNV involving part of the long arm of chromosome 4 in a phenotypically normal mother and child. The identification of this case contributes to additional understanding of deletion at 4q32.2q32.3. This report may provide a reference for prenatal diagnosis and genetic counselling in patients who have genotypes of similar cytogenetic abnormalities.

**Conclusions:**

The novel 7.22-Mb deletion at chromosome 4q32.2q32.3 (162858958-170081268) is a VUS. The foetus inherited this VUS from a phenotypically normal mother.

## Background

Chromosomal microarray analysis (CMA) is currently widely used in prenatal diagnosis. It mainly has two technology platforms: array-based comparative genomic hybridization (aCGH) and single nucleotide polymorphism array (SNP array) [1]. Although CMA is a powerful diagnostic technology for detecting chromosomal copy number variants (CNVs) [2], it detects numerous variants of unknown significance (VUSs). These VUSs pose a great challenge for genetic counselling. Typically, a VUS is a rare or novel CNV in that is not known to have correlation with a clinical disease, and its pathogenicity can neither be ruled out nor confirmed. Based on the existing literature and databases, a VUS may be classified as “likely pathogenic”, “uncertain significance” or “likely benign” [3]. Parental verification testing can assist with classification because it can determine whether the variant occurred as a de novo or inherited genetic mutation in the foetus.

Terminal deletion of the long arm of chromosome 4 is a rare genetic aberration with an estimated prevalence of 1:100,000 [4]. Clinically, this kind of genetic aberration is known as “chromosome 4q-syndrome”. The common phenotypic features of 4q-syndrome include limb abnormalities, cardiac malformations, mental retardation, developmental delay, dysmorphic facial anomalies, Pierre Robin sequence and digital anomalies [5, 6]. In 1967, Ockey et al. first reported a deletion of the long arm of chromosome 4 in a child with limb abnormalities [7]. Since then, hundreds of patients with chromosome 4q-syndrome have been reported [4, 8–11]. The severity of the phenotype is correlated with the size of the deleted ranges (larger or smaller). It has been reported that del(4)(q32q33) has mild to moderate clinical symptoms [12]. Usually, deletions involving 4q31 determine more severe malformations than deletions involving band 4q34 [13].

In this case, we present a novel VUS of a 7.2-Mb deletion at chromosome 4q32.2q32.3 and illustrate the importance of reporting unusual variant chromosomes for genetic counselling purposes.

## Case presentation

A 31-year-old healthy pregnant woman, gravida 2 para 1, underwent non-invasive prenatal testing (NIPT) at 15 weeks of gestation at Xingtai local hospital. NIPT was performed as previously described [14]. The result of NIPT indicated a 7.35 Mb deletion at chromosome 4q32.2q32.3(162582601-169932600del). There are several possible reasons for this result: chromosomal abnormalities in the mother, chromosomal abnormalities in the foetus, and chromosomal abnormalities in the placenta. The woman was then referred to our hospital at 22 weeks of gestation for prenatal diagnosis and genetic counselling. Her family and previous histories were uneventful. Her pregnancy history resulted in the term birth of a healthy boy. The sonographic examination did not reveal any ultrasound anomalies. After being informed about the possible risk, the woman decided to undergo amniocentesis. Prenatal karyotyping and CMA techniques were subsequently performed.

## Methods and results

**Fig. 1:**
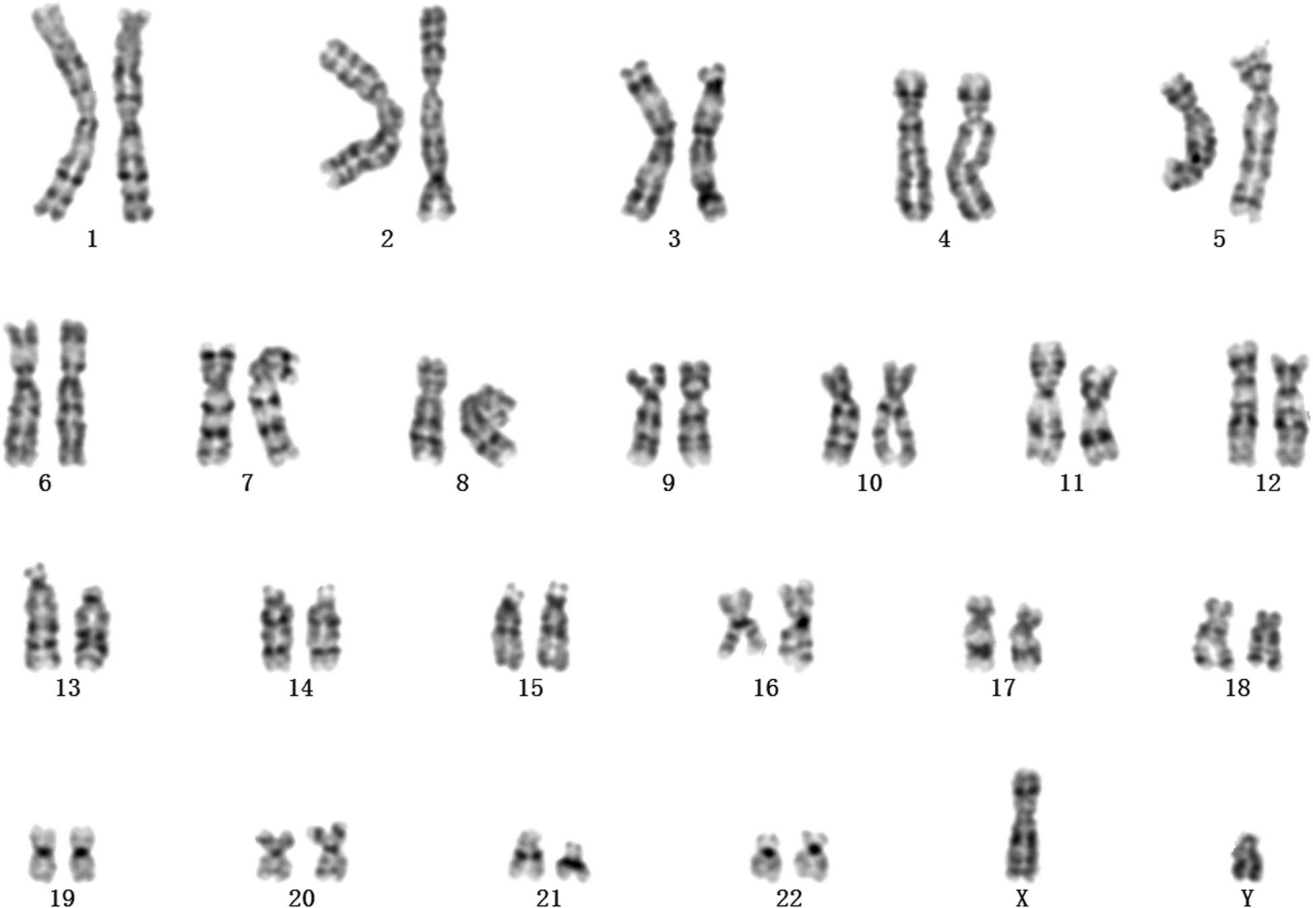
G-banded karyotype of the foetus indicated an apparently normal karyotype of 46, XY

Chromosome analysis was carried out on cultured cells obtained from amniotic fluid by conventional Giemsa-band karyotyping at approximately 320-band resolution. The cytogenetic analysis revealed an apparently normal karyotype of 46, XY (Fig. 1) with limited banding resolution.

**Fig. 2:**
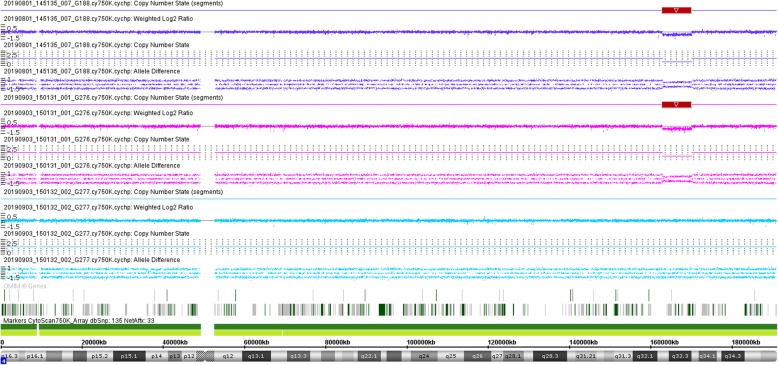
SNP array analysis showing that the paternal chromosomes were normal, while the foetus and the mother had the same 7.22-Mb deletion at 4q32.2q32.3. (The color of purple represents the result of the foetus. The color of pink represents the result of the mother. The color of light blue represents the result of the father. The red block indicates the position of the 4q32.2q32.3 deletion.)

Microarray-based copy number analysis was performed using the Chromosome Analysis Suite software version 4.0.0.385 (R28959) (Thermo Fisher Scientific Inc.) and the results were presented on the International System for Human Cytogenomic Nomenclature 2016 (ISCN, 2016). To interpret the results of the SNP array, we used the Database of Cases with Heteromorphisms (http://ssmc-tl.com/HMs.html), Decipher Database (DECIPHER, http://decipher.sanger.ac.uk/), the Database of Genomic Variants (DGV, http://www.ebi.ac.uk/dgva/), Online Mendelian Inheritance in Man (OMIM, http://omim.org/), Clinical Genome Resource, (Clingen, https://www.ncbi.nlm.nih.gov/projects/dbvar/clingen/) and PubMed (http://www.ncbi.nlm.nih.gov/pubmed/) to determine the clinical significance of CNVs. CMA using the Affymetrix CytoScan 750 K SNP microarray (Affymetrix CytoScan 750 K Array, Santa Clara, California), was performed on DNA extracted from amniotic fluid and a 7.22-Mb deletion was detected at chromosome 4q32.2q32.3 or arr[GRCh37] 4q32.2q32.3(162858958_170081268)×1 (Fig. 2). SNP array analyses of the parental blood showed that the paternal chromosomes were normal, but the maternal chromosomes had exactly the same deleted region as the foetus at chromosome 4q32.2q32.3 (Fig. 2).

After genetic counselling, the couple decided to continue the pregnancy and a male neonate weighting 3050 g was born by caesarean section on November 12, 2019, with a five-minute Apgar score of 10 points, and no abnormal clinical symptoms or signs have been observed to date.

## Discussion

In this case, the G-band karyotyping analysis of the foetus indicated an apparently normal karyotype of 46, XY (Fig. 1). As a gold standard procedure, conventional Giemsa-band karyotyping on metaphase cells can detect chromosomal aberrations at a resolution of 5–10 Mb [15]. Thus, deletions or duplications that are smaller than 5–10 Mb may be overlooked unless additional techniques are used [16]. CMA can provide much greater resolution and potentially detect CNVs and differences in the amount of chromosome samples as small as 100 to 200 kb [1]. The SNP array analysis of uncultured cells obtained from amniotic fluid showed a result of arr[GRCh37] 4q32.2q32.3(162858958_170081268)×1 (Fig. 2). This result is almost consistent with the previous NIPT result. Unexpectedly, no benign reports about this loss were found in the DGV database; no pathogenicity was reported in Decipher Database; and no dose sensitivity was reported in the Clingen database.

A total of 47 genes (including 15 OMIM genes) with already known or unknown functions have been mapped to the 7.22-Mb deleted region of chromosome 4q32.2q32.3(162858958-170081268). This region encompasses 3 protein coding and pathogenetic OMIM genes named *Carboxypeptidase E (CPE)* [MIM 114855], *Tolloid Like 1 (TLL1)* [MIM 606742] and *Palladin, Cytoskeletal Associated Protein (PALD)* [MIM 608092]. According to the Online Mendelian Inheritance in Man (OMIM, http://omim.org/), *CPE* is responsible for maturity-onset diabetes of the young, type 6 and hyperproinsulinaemia; *TLL1* is involved in atrial septal defect 6 and atrial septal defect ostium primum; and *PALLD* is associated with pancreatic cancer 1 and pancreatic cancer. Other related genes in the 7.22-Mb deleted section may also contribute to the variable features, especially *Methylsterol Monooxygenase 1 (MSMO1)* [MIM 607545], which is known to be associated with diseases including congenital cataract, microcephaly and psoriasiform dermatitis.

In the existing literature, more than 100 cases have been previously reported on 4q deletions [4, 8–11]. However, no cases had interstitial deletions sharing the same deleted segment as the present case. To date, only a dozen cases involving the affected region of 4q32 have been reported (Table 1). In 1992, an 18-month-old girl with a cardiac defect, duplicated kidney, and postnatal growth failure was reported to have a distal deletion of 4q3l .22-q34.2, detected by using high resolution G banding on lymphocytes [17]. The genotype of del(4)(q32q34) is the most common 4q deletion and contributes to mild to moderate clinical symptoms including developmental delay [19–21] and congenital heart defects [23]. The most systematic research was performed by Strehle E M et al. [11]. They characterized 20 patients with 4q deletion syndrome by using array CGH and compared the clinical characteristics found in these patients with those of the 101 patients reviewed by Strehle and Bantock [4]. In addition, a 6-month-old boy presenting with congenital heart disease and clenched hands was reported with an interstitial deletion at 4q31-q32 [13]. More recently, Tidrenczel Z et al. [9] reported a prenatal diagnosis of a foetus with a 33.5 Mb deletion of 4q32.1-q35.2 presenting with high-risk combined screening test results and second trimester ultra-sound soft markers. Aladhami S M S et al. [18] reported a maternal inherited del(4)(q32q33) which were not leading to major malformations in affected persons. A 12-year-old proband and his 29-year-old mother both showed mild dysmorphic features, obesity, late presentation of learning difficulties and behavior problems [18]. This case lost the whole region of 4q32 and 4q33 (approximately 18 Mb large) which comprised the affected region of our case (7.22-Mb deletion at 4q32.2q32.3) completely. Generally, the smaller the deletions are the milder are the phenotypes as compared with larger deletions. Interestingly, a 22-year-old individual reported by Tzschach A et al. [22] with mild to moderate mental retardation, psychosis and obesity was found to have a 4q32.1-q32.3 deletion, which comprised a deleted region the most similar to our case but smaller than the case del(4)(q32q33) discussed above. According to Tzschach A et al. [22], the 10-Mb deletion at 4q32.1q32.3 was harbored more than 30 genes, and haploinsufficiency of one or several of these genes is likely to have caused the clinical problems of the patient. The above patients possessed distal 4q deletions that overlapped with our case, but no individuals with a reported interstitial deletion were identical to the deletion found in the proband.

**Table 1:** Summary of genotype-phenotype correlation on the affected region of 4q32

Author	Age/Sex	Deletion regions, start-end	Deletion size (Mb)	Clinical features
Sarda P et al.,1992 [17]	18 Month/F	del(4)(q3l .22q34.2)	–	A cardiac defect, duplication of left kidney, skeletal abnormal, postnatal growth failure.
Aladhami S M S et al.,2000 [18]	12 Year/M 29 Year/F	del(4)(q32q33)	–	A 12-year-old boy showed mild dysmorphic features, late presentation of learning difficulties and behaviour problems, obesity, breast hypertrophy and bilateral slip-ped capital femoral epiphysis. His mother also has mild dysmorphic features, obesity, and a similar history of late presentation of learning difficulties and behaviour problems.
Keeling S L et al., 2001 [19]	An infant	del(4)(q32q34)	–	Mild developmental delay; a left ulnar ray defect with absent ulna and associated metacarpals, carpals and phalanges; and a right ulnar nerve hypoplasia.
Ramanathan et al., 2004 [20]	11 Year/M	del(4)(q32q34)	–	Early developmental delay and minor dysmorphic features.
Kaalund et al., 2008 [21]	7 Year/M	del(4)(q32.1q34.3)	–	Respiratory problems, developmental delay, learning difficulties, bilateral ptosis, low set ears and anteverted nares, prominent cheeks, micrognathia, small and open mouth, macroglossia, and teeth abnormalities.
Tzschach A et al., 2010 [22]	22 Year/F	4q32.1q32.3	10 Mb	Mild to moderate mental retardation, psychosis, obesity, broad nasal root, sparse lateral eyebrows, thin upper lip, short philtrum, micrognathia, and strabismus.
Ismail S et al.,2012 [13]	6 Month/M	del(4)(q31q32)	–	Congenital heart disease and clenched hands.
Strehle E M et al., 2012 [11]	13 Year/M	Chr4:16407495–188987971	24.9 Mb	Facial asymmetry, glabellar hemangioma, prominent nasal root with hypoplastic alae, short nose with anteverted nares, overfolded ear helices, flat philtrum, cleft soft palate, dental crowding, fine long hair under chin.
Strehle E M et al., 2012 [11]	4 Year/F	Chr4:164807106–190490075	25.7 Mb	Hypoplastic supraorbital ridges, large fontanelles, upslanting and shortpalpebral fissures, hypertelorism, glabellar hemangioma, overfolded ear helix, microstomia and micrognathia.
Strehle E M et al., 2012 [11]	5 Year/F	Deletion:166719262–4qter; Duplication:705175–20pter	24.6 Mb	Increased fetal nuchal translucency, microcephaly, broad nasal bridge, full cheeks, absent lower incisors, cleft palate, micrognathia.
Strehle E M et al., 2012 [11]	2 Year/F	166860495–4qter	24.5 Mb	Epicanthic folds, upturned nose, receding chin.
Xu W et al.,2012 [23]	8 Month/−	del(4)(q32.3q34.2) Chr4:167236114–178816031	11.6 Mb	Congenital heart defect.
Tidrenczel Z et al., 2019 [9]	A Fetus/M	del(4)(q32.1q35.2) Chr4:157455107–190957460	33.5 Mb	High-risk combined screening test results and second trimester ultra-sound soft markers.
Present Case	31 Year/F and her child	del(4)(q32.2q32.3) Chr4: 162858958-170081268	7.22 Mb	Normal phenotype.

Based on what has been discussed above, the 7.22-Mb deletion of chromosome 4q32.2q32.3 is a VUS. Its pathogenicity cannot be ruled out definitely. The VUS in prenatal diagnosis usually pose a great challenge for genetic counselling. The information obtained from prenatal diagnosis could facilitate prospective parents’ reproductive decision-making when confronted with the choice between terminating pregnancy and continuing pregnancy. How do we avoid ending a potentially benign life; but avoid the pain and suffering that a defective child may bring to a family? This is not easy to answer when encountering VUSs clinically. Most women wish to be reassured that their unborn child is healthy. Inevitably, any prenatal diagnosis programme that aims to provide such reassurance will cause anxiety, especially those with diagnoses of unknown clinical significance. Beneficial and clear counselling may ease the anxiety of a pregnant woman and reduce the chance of medical disputes.

To further clarify the clinical significance of the proband, parental verification tests are subsequently required. SNP array analyses of the parental blood showed that the paternal chromosomes were normal, while the maternal chromosomes had exactly the same deletion region at chromosome 4q32.2q32.3 compared with that of the foetus (Fig. 2). Usually, when a mother and her unborn baby carry the same VUS at an autosome, the baby is less likely to be pathogenic if the mother is healthy and normal. To the best of our knowledge, this is the first case report on a VUS of 4q32 deletion and the second report of a CNV involving part of the long arm of chromosome 4 in a phenotypically normal mother and child. Docherty Z et al. first reported a prenatal case of a rare heterochromatic variant on chromosome 4 in 1984. A phenotypically normal foetus inherited the abnormal karyotype of 46,XY,add(4)(q35)? from a clinically healthy woman [24].

## Conclusion

The novel 7.22-Mb deletion at chromosome 4q32.2q32.3(162858958-170081268) is a VUS. The foetus inherited this variant from an asymptomatic and healthy pregnant woman without any ultrasound anomalies. It appears that the 7.22-Mb deletion is a rare heterochromatic variant. After genetic counselling, the couple decided to continue the pregnancy and a male neonate with a normal phenotype was born at 39 plus 3 weeks of pregnancy.

## Data Availability

NA

## Abbreviations

CMA: Chromosomal microarray analysis
SNP array: Single nucleotide polymorphism array
aCGH: Array-based comparative genomic hybridization
Mb: Megabasepairs
UPD: Uniparental disomy
CNVs: Copy number variants
NIPT: Non-invasive prenatal testing
VUSs: Variants of unknown significance
ISCN, 2016: the International System for Human Cytogenomic Nomenclature 2016
